# Gun-Shot Wounds—Army of Northern Virginia

**Published:** 1864-10

**Authors:** F. Sorrel

**Affiliations:** Surgeon and Inspector of Hospitals.


					CONFEDERATE STATES
MEDICAL & SURGICAL
OURSIAL.
Vol. I. RICHMOND
OCTOBER, 1864
No. 10.
0 RIG 1NAL COM MI 'NIC ATIOXS.
Art. I-
Gun-Shot Wounds?Army of Northern 17rglnia.
[An extract mm a lleport on the Sickness and Mortality
in the Armies of the Confederate States for 1863.]
Bv
F. Sorrel, Surgeon aud Inspector of Hospitals.
GUN-SHOT WOUNDS. 3
The proportion of these was less than that of last year by
2 per cent.; that is to say, while gun-shot injuries, during
the period covered by the report of last year, amounted to 9.8
of the whole number of cases reported; for 18G3, they ouly
reach 7.7. It will be remembered that the campaign of
18<j2 was conducted with the utmost vigor, and that the
army fought, in quick succession, many sanguinary battles,
commencing with that of Williamsburg and ending with that
of Fredericksburg; including between these two the battles
arouud Richmond, Cedar Mountain, Manassas and Sharps-
burg, each of which resulted in a heavy list of casualties to
the troops engaged.
The mortality from these causes, as might be expected, was
not nearly so great during this year as it had been during the
one previous. The mnn had become hardened and better able
to resist the influence of climate and exposure, and the bat-
tles, which occurred during the year, transpired at a season
far more favorable to recoveries. Thus, instead of being
crowded together in hastily extemporized hospitals during
the intense heats of mid summer, as they were in 1862, the
battle of Chancellorsvillej fought early in May, gave to its
wounded the benefit of a most delightful and salubrious season
for treatment and recovery, while that of Gettysburg, still
later, sent ouly to our hospitals the more slightly wounded,
leaving the grave cases in the hands of the enemy to die, or
to be otherwise accounted for iu a manner not known to our
reports.
The surgical operations performed on uie lieid at Ubancel-
lorsville did remarkably well. Generally, the wounded
reached the hospitals in this city in a condition favorable to
recovery, and as before stated, the excellent condition ol our
hospitals seemed to repress all tendency to the prevalence oi
erysipelas or gangrene. Indeed, never before or since have
they been so entirely free from the presence of these diseases,
as they were during the summer aud fall ot this year.?
12
In the hospitals, the mortality from wounds, during the
year 1862, amounted to 11.2; in 1863, to 2.3; and this dif-
ference is really increased still further in favor of the latter
year, by the fact, that the wounded from the battle of Frede-
ricksburg gave most of its mortality to the year 1863, with-
out addiug at all to the number of cases for that year?these
having been already embraced in reports for 1862.
The figures, as wc have them in our reports for this year,
exhibit 42,885 cases of Wounds treated in hospitals, and 999
deaths?this yields a mortality of 2.3. But, in order to ar-
rive at a more accurate estimate of the proportion really dy-
ing from wounds in hospital, it will be well to assume that
the number reported from the field were all that were treated
in hospitals; and this being 27,200, and the mortality 999,
we have a per centage of 3.7, which, it is .believed, approaches
nearer the truth.
Adding the number dying in hospitals to those reported1
from the field, we have an aggregate of deaths for the year
in field and in hospital, from guu-shot wounds, of 1,728, or
2.4 of the whole number of cases reported.
This, however, is much too small, because it is impossible,
as before stated, with our present form of reports, to avoid the
frequent multiplication of cases by "transfer, &c.
])uring this year, one case of successful amputation at the
hip-joint was reported. It occurred in the persou of James
Kelly, private company " B," oGth Pennsylvania, aged 28
and by occupation a farmer. He was wounded April 29th,
1863, near Fredericksburg, sustaining compound comminuted
fracture of the femur. Disarticulation, by antero-posterior
flaps, was performed the same day on the field He fully re-
covered, was paroled and sent North July, 14th, 1863.
( Operation -performed by Surgeon E Shippen, U. S. A.)
It is proper to remark, also, in this connection, the many
successful cases of amputation, especially of the upper-third
of the thigh, occurring in our reports. Among them may he
mentioned Lieutenant-G-enerals Ewell and Hood, (the latter
now General Commanding Army of Tennessee,) both of whom
have been restored to duty, in the full vigor of health, with
thighs amputated just a little short of the hip-joint. Added
to these are many others, of less distinction, it is true, but
not the less attesting the skill and ability of Confederate Sur-
geons in the performance of an operation, regarded heretofore
as almost uniformly fatal. Indeed, so much had this come to be
regarded the case, that recently an order emanating"from the
154 CONFEDERATE STATES MEDICAL AND SURGICAL JOURNAL.
V**ra&r? T' jh ||M|| n, pi mminili??i|i||||?|fM?? lilllWITlUTTTI III |i|H||| ilillM mmillBI'l ml Ml
Federal Surgeon-Geuer I forbids such operations on the field,
not only at the upper-third, but anywhere along the continuity
of the thigh.
This question of Conservative Surgery in compound frac-
tures of the femur, is one which is receiving the most earnest
attention of military surgeons, and though it has been, to some
extend, decided in the I nited States, we Sire obliged, in bal-
ancing the merits of the two methods of treatment, to adopt
a different opinion. With us the results, as elicited from our
r port-?, xhibit a slight pev centagc in favor of the operation
itself on the field. Thus, of 77 cases of primary amputation
of :Jr t: ' h, at the upper-third, 40 recovered, and 37 died.
F]"? me '' le ?-???-:Its are also found to have attended the
e ??? * letLev of treatment in simitar cases: for, of 2:21
cases, wh' re amputation was not resorted to, TIG recovered.
These results are indeed remarkably favorable; far surpass-
ing, in this way, any heretofore reported or kuown to the
profession.
As between the two modes of treatment, the difference in
the results is but slight, as shown by the figures given above,
and as will be more perfectly understood when the tables an-
nexed shall 1 ex mined Guided, then, by our reports, it
may be safely accepted as a rule, that the better plan, in
general, is to operate on the field. The greater readiness with
which the patient can be transported from the field; the
greater ease and comfort realized under these circumstances,
when the limb has been removed; the lesser time required in
hospital for recovery, would all seem to point to its adoption
as the wiser policy. Still, in these and in all other questions
of surgical interference, the medical officer should be governed
by the peculiar circumstances attending each case.
Many resections were performed during this year; and while
in very many instances with results altogether favorable, so far
as recovery alone is considered, yet, we are inclined to think,
in nearly all cases, leaving limbs of very doubtful utility.
Indeed, when it is considered that these operations arc much
more fatal than simple amputations, exposing the patient for
a much greater time to the evil influence of hospital atmos-
phere; involving more frequently, too, attacks of erysipelas,
gangrene, nod pyemia, it may be well questioned if our sur-
geons do not too often resort to them.
The shoulder-joint, we sometimes think, is the only one in
which resection promises all the good results claimed for it.
In the elbow, if the entire joint be removed, no possible effort
of nature can supply the lost motion; and the slight prehen-
hie power of the lingers, which mny continue, can scarcely
atone for the awkWard, Useless and ungainly limb remaining.
Exsection of the knee joint we cannot help regarding as
positively reprehensible on the field, and scarcely less so in
hospital. But one succesafu\ ease is reported during the
year, (Surgeon J. 13. Read;) at last accounts, (September,
186*,) the patient was fully recovered, but with a limb short-
ened by several inches, and union only ligamentous. Ampu-
, tation has been asked for by him, and will be performed as
soon as the condition of his health may jnstify it.
One successful case of resection of hip-joint has likewise
been reported by .Surgeon J. B. Head, and though the lej
will never be of any use, yet it is fair to say, that to this ope-
ration may be due the preservation of the patient's life, which
might have been more seriously imperilled by disarticulation.
Frequent cases of gun-shot wounds, healing by first inten-
tion, have also been reported. It is difficult to understand
how this can be so, when all our theories of repair and resto-
ration of lost tissues have been based on inflammatory action,
leading, of course, to suppuration and granulation. Still, the
evident care and truthfulness which accompany the report of
the cases attest the fact beyond all doubt; and it is hoped
that future investigations on the part of the Medical Staff will,
in time, yield much that is interesting and instructive in this
connection. Already, it has been proposed (by Surgeon J. J.
Chisolm) to convert all gun-shot wounds into simple incised
wounds, by paring the ragged edges, and nicely adjusting the
lips by means of sutures or straps, excluding the atmosphere,
and thus effecting a cure by absorption and re-modelling, with-
out the aid of suppuration.
TETANUS.
In reviewing carefully all the circumstances connected with
gun-shot wounds, as exhibited in the reports before us, it
would be singular did we not remark the occasional occurrence
of tetanus.
This complication of gun-shot wounds, so obscure, so fear-
fully fatal, and so much to he deplored, is fortunately seldom
met with. Indeed, it has always been a source of wonder to
all writers ou military surgery that it so rarely occurs. In our
reports for 1861, we find that in 1,750 cases of wounds of
different characters, t here were thirteen cases of tetanus?0.75,
or one case of tetanus in 131 cases of wounds. Of these only
three are reported as having ended fatally, giving a ratio of
mortality of 2.3, or one death in four eases: a result which
clearly proves the inaccuracy of our earlier reports.
In 1862, the consolidated reports from hospitals present
15,971 cases of wound*, uud only 53 cases of tetanus?0.11,
or one case of tetanus ia 867 cases of wounds. Of these 53
cases, 28 terminated in death?52.8, about one death in two
cases, a much larger"per centage of mortality than in 1861?
DUE stilly we nave reason to suspect, very much below the
truth. Many ol the cases reported tetanus are, doubtless,
mere cases of traumatic spasms, hence such apparently favor-
able results.
Tetanus was doubtless of more frequent occurrence in our
hospitals than would appear from the reports, as it is well
known that cases are generally reported under the disease
they first enter with, which ordinarily is ""V ulnus Sclopeti-
cum." It is only reccutly that surgeons have been reporting
"supervening diseases." In referring to our special reports of
gun-shot injuries involving tetanus, covering the whola period
of the war up to the present time, aud which are evidently
drawn up with great care, we hod 66 cases recorded. Of
these, six only recovered, giving a mortality of nearly 91 per
cent,
Assuming, then, that these constitute the entire number of
cases of decided tetanus which have occurred on the whole
CONFEDERATE STATES MEDICAL AND SURGICAL JOUJRNAL.
number ci' gun-shot wounds treated, (50,775,) we have the
proportion of 1 case of tetanus to every SCO cases of gun-shot
wound.
McLood, in Lis Surgery of the Crimean War, does not give
the proportion of tetanus to wounds, but he says he could only
hear of thirteen casts of tetanus in the English army through-
out the Crimean war. These were all fatal?with only one
exception. In the last East India war, nineteen cases are re-
ported, and only one recovery. Alcock gives the proportion
of cases of tetanus to wounds as one in sevcnty-niue. Strom-
yer states, that during the Schleswig-IIolstein war, the pro-
portion was ,3 in 1,000. In 1830, of 390 cases of gun-shot
wound, there was only one case of tetanus in the Hotel Dieu
at Paris.
The following tables which exhibit the general results of am-
putations and resections thus far collected, and carefully pre-
pared from reports throughout the Confederacy:
Primary?
Successful
Unsuccessful.
Secondary?
Successful
Unsuccessful.
Useful Joints
Total
RESECTIONS
Wrist? Hip. | Knee.
Shoul'r.
28
13
20
7
2
Elbow.
22
3
23
C
7
70. | G1
Primary?
Case3
Deaths...
Secondary-
Cases
Total.,
D ISABTIC ULATIONS.
Siioul'r.
Death3  20
54
25
9
108
Elbow. Wrist. Hip. Knee.
1 2 ; t>5
2 3 33
11
*> 27
!
11 136
AMPUTATION 04" THTGH.
Circular?
Primary
Secondary
Flap?
Primary
Secondary
Method not stated-
Primary.
Secondary.
Total.
T ?
U P P E 11 j M I D D L K \j 0 W K R '
Third, i Third. ; Third.
COMPOUND FRACTURE OF THE THIGH TREATKf> WITIhit V
A MP I TAT ION.
Average Period of Recovery.,
Greatest Period of Recovery.
Least Period of Recovery
Average Period of Reath.
Greatest "Period of T)eath.
Least Yeriod of {3eatb
Average Amount of Shortening.
Greatest "^Amount of Shortening.
Least Amount of",Shortening
116
105
CONSOLIDATED TABLE OF AMPUT VTIONP.
Primary. Secondary.
Thigh  345 ,213 122
Leg '::14 210 or.
Aria   204 _'52 42
Fore-Arm  69 GI 8
Shoulder-Joint \ T9 | 54 25
Elbow-Joint    4 3 1
Wrist-Joint  7 5 2
Hip Joint  :: 1 2
Kuee-Joint 3
AnkleJoint i G 1
Taraal-Joint 1 16 13
Total  1142
827 315
38 I1G2
30 pl50
14 i140
12 | 45
31 ! 28
25 i1 3
28 ...
6*6 ...
GO jj 6
33'| 4
10 | 8
! |
27 54G
43
7G
87
C
119
74
53
10
20
1
... ! (, 100
4
262 284 I 51

				

